# Development of a Self-Reported Measure of Academic Pressure Among Secondary-School Students: The Academic Pressure Questionnaire

**DOI:** 10.1177/13591045261430414

**Published:** 2026-03-11

**Authors:** Marie A. E. Mueller, Chris Bonell, Tamsin J. Ford, Carolina Gutiérrez Muñoz, Ann John, Glyn Lewis, Rebecca Meiksin, Simon Murphy, George Ploubidis, Ruth Ponsford, Frances Rice, Thomas Steare, Alice Sullivan, Neisha Sundaram, Nerissa Tilouche, Gemma Lewis

**Affiliations:** 1 Division of Psychiatry, Faculty of Brain Sciences, 4919 University College London, London, UK; 2 Department of Public Health, Environments and Society, London School of Hygiene and Tropical Medicine, London, UK; 3 Department of Psychiatry, 2152 University of Cambridge, Cambridge, UK; 4 Department of Psychology, 1555 University of Bath, Bath, UK; 5 National Centre for Suicide Prevention and Self-Harm Research, Swansea University Medical School, 7759 Swansea University, Swansea, UK; 6 Centre for the Development, Evaluation, Complexity and Implementation in Public Health Improvement (DECIPHer), School of Social Sciences, 2112 Cardiff University, Cardiff, UK; 7 Wolfson Centre for Young People’s Mental Health, 2112 Cardiff University, Cardiff, UK; 8 Centre for Longitudinal Studies, Social Research Institute, 4919 University College London, London, UK; 9 Centre for Neuropsychiatric Genetics and Genomics, Division of Psychological Medicine and Clinical Neurosciences, 2112 Cardiff University, Cardiff, UK; 10 Unit for Lifelong Health and Ageing at UCL, UCL, London, UK

**Keywords:** academic pressure, mental health, schools

## Abstract

**Purpose:**

There is evidence that academic pressure has been rising among adolescents in the UK. While this may be a modifiable risk factor for mental health problems, there are few validated measures of academic pressure and all have limitations.

**Methods:**

With secondary-school students, we co-produced a student-reported measure of academic pressure, the 7-item Academic Pressure Questionnaire (APQ). This was included in the baseline survey of students aged 12-13 within the Positive Choices trial, a whole-school intervention to promote sexual health in English secondary schools. We ran factor analyses and assessed internal consistency, associations with sex and depressive symptoms, and variation in academic pressure between schools.

**Results:**

We extracted one factor (Cronbach’s alpha 0.76). Female students had higher APQ scores than males (mean difference = 2.18, 95% CI: 1.88 to 2.49). Higher APQ scores were associated with more depressive symptoms (coefficient = 0.51, 95% CI: 0.48 to 0.55) and associations were larger in female than male students (p value for interaction <0.001). School-level factors explained 2.6% of variation in APQ scores after adjusting for individual-level factors (ICC = 0.026, 95% CI: 0.01 to 0.06).

**Conclusion:**

The APQ is a valid and reliable tool to investigate academic pressure in secondary-school adolescents.

## Plain Language Summary

Pressure to do well at school has been rising among teenagers in the UK, and may increase the risk of mental health problems. There are few questionnaires available to measure school pressure among secondary school students and none have been produced in consultation with young people in the UK. Together with young people from Wales (UK), we developed a questionnaire to measure school pressure among secondary-school students; the Academic Pressure Questionnaire (APQ). We included the APQ in the Positive Choices study of an intervention to promote sexual health among 12- to 13-year-olds from 50 schools in England. Our study found evidence that the APQ was reliable and valid. Female students experienced higher levels of school pressure than male students. Students with higher levels of school pressure also reported more symptoms of depression, and this association was stronger in female than male students. Levels of school pressure varied between schools, although by a relatively small amount. The APQ appears to be a useful new tool for measuring school pressure among secondary school students.

## Introduction

Academic pressure has been rising among adolescents in the UK and may be a modifiable risk factor for mental health problems (Armitage et al., 2025; Steare et al., 2023). There is no standard definition of academic pressure, which has made measurement challenging. However, it can broadly be defined as adolescents’ perceptions of the demands of their schoolwork, and the responsibility, expectations and importance of educational performance (Asgarabad et al., 2021; Högberg, 2021). There are few validated measures of academic pressure, all of which have limitations. Many use single items while most multi-item scales include too many questions to be routinely included in large surveys. Most scales were not co-produced with young people in terms of defining academic pressure and developing items. No multi-item scale has been co-produced with young people in the UK.

We previously conducted patient and public involvement and engagement (PPIE) with young people in Wales (UK) to co-produce a definition of academic pressure. Consistent with theory and research (Steare et al., 2023), young people stated that the important components of academic pressure were fear of failure, concerns about the future, high workload and exams, worries about parental and teacher expectations, and competition with peers for grades.

In this study, with young people in Wales (UK), we co-produced a measure of academic pressure, the Academic Pressure Questionnaire (APQ). To assess validity, we investigated differences in academic pressure between females and males (expecting females to score higher than males) (Steare et al., 2023). We then investigated cross-sectional associations between the APQ and depressive symptoms (expecting higher APQ scores to be associated with more depressive symptoms and for this association to be stronger in females than males) (Steare et al., 2023). We also examined differences in academic pressure between schools (expecting schools to differ in their academic pressure scores). We chose these measures of validity based on existing literature and theoretical assumptions.

## Methods

Our Statistical Analysis Plan was pre-registered (https://doi.org/10.17605/OSF.IO/PKC8V).

### Item Development

We co-produced the APQ for secondary-school students (aged 11–18) with the Advice Leading to Public Health Advancement (ALPHA) group of young people at the DECIPHer Centre in Cardiff. ALPHA consists of approximately 20 young people aged 14–25 years who are trained to provide advice on public health research. We conducted two 1.5-hour-long online workshops (with 20 young people; 16 females and 4 males; other demographics not reported for anonymity) to refine the definition of academic pressure and the APQ questions. Based on young people’s definition, theoretical perspectives, and existing measures (Table S1), we created a draft measure of 10 items (Table 1). In workshop 2, young people commented on the readability, clarity, and relevance of items. We modified items based on this input, removing three (Table 1). The item ‘My school sets too much homework’ was removed because young people reported that most would agree. The item ‘I’m confident I will live up to my academic standards’ was removed because young people stated they did not understand it. The item ‘My grades are important to my future and might even determine my whole life’ was removed because young people could not relate to this. This resulted in a 7-item questionnaire which we used in our main analysis. We ran a supplementary analysis using all ten items.

**Table 1.** Items of the APQ

Competition with peers for grades is intense.
My parents’ expectations about grades put me under pressure.
There is pressure from teachers to perform well in tests and exams.
I worry about doing well in tests or exams.
My school sets too much homework.^a^
I have too many tests and exams.
I’m confident I will live up to my academic standards.^a^ ^(rs)^
If I fail to do well in school, I’m a failure as a person.
My grades are important to my future and might even determine my whole life.^a^
Even if I do well in school, I’m worried about getting a job in the future.

*Note.* Response options range from 1 ‘strongly agree’ to 5 ‘strongly disagree’.

^a^ These items were removed after additional feedback from the Advice Leading to Public Health Action (ALPHA) group. The items, ‘My school sets too much homework’, ‘I’m confident I will live up to my academic standards’, and ‘My grades are important to my future and might even determine my whole life’, were removed after consultation with young people. Items are recoded as: 1 = 4; 2 = 3; 3 = 2; 4 = 1; 5 = 0. ^(rs)^ This item is reverse scored. The total APQ score is the sum of the items. The total APQ score ranges from 0 ‘low academic pressure’ to 28 ‘high academic pressure’ for the 7-item version of the APQ.

### Sample

The APQ was included in the baseline survey of the Positive Choices trial of a whole-school intervention to promote sexual health among secondary-school students in England (Ponsford et al., 2021, 2022). Fifty secondary schools were recruited from across southern and central England between March and July 2021. Recruitment was through emails and phone calls to schools, local authorities, school networks and academy chains. A total of 6,970 students aged 12–13 participated in baseline surveys between November 2021 and March 2022 (Ponsford et al., 2021, 2022).

### Measures

The seven APQ items were recoded and summed (total scores ranged from 0–28). Higher scores indicating higher levels of academic pressure.

Depressive symptoms were assessed with the 8-item Patient Health Questionnaire, PHQ-8 (Kroenke et al., 2009), a modified version of the 9-item PHQ (PHQ-9) (Kroenke & Spitzer, 2002). Total scores ranged between 0 and 24, higher scores indicating more severe symptoms.

Sex was measured with a binary variable (female/male). Ethnicity was measured with six groups: ‘Asian or Asian British’, ‘Black African, Black Caribbean or Black’, ‘Mixed/multiple ethnic groups’, ‘White’, and ‘Any other ethnic group’.

We selected a set of potential confounders based upon theoretical assumptions and existing evidence (see supplement, page 2, for details on measurement).

### Statistical Analyses

#### Factor Analysis

We ran exploratory factor analysis (EFA) and confirmatory factor analysis (CFA) to identify the factor structure (see supplement pages 2–3 for details on methods). We randomly divided our sample into two, using the first sample for EFA and the second for CFA.

#### Reliability

We calculated Cronbach’s alpha to assess internal consistency for each factor extracted.

#### Validity

We maximised content validity by co-producing the APQ with ALPHA. We could not assess criterion validity because there is no gold-standard measure. We could assess construct validity to some extent by investigating associations of the APQ with sex and depressive symptoms, as well as variation in APQ scores between schools. We also explored whether APQ scores differed according to other student and school characteristics (these analyses were not pre-registered).

We compared APQ scores between female and male students by running a multilevel linear model with APQ scores as outcome, sex as exposure, and a random intercept for school.

In analyses of the association between academic pressure (exposure) and depressive symptoms (outcome), we described characteristics of the sample overall and according to levels of academic pressure (mean [SD] for continuous variables, number [%] for categorical variables). For descriptive purposes only, we used a binary version of the academic pressure variable split at the median. We then used multilevel linear regressions with a random intercept at the school level, before and after adjusting for potential confounders. In fully adjusted models, we tested whether the association was stronger in female than male students by calculating an interaction between exposure and sex.

We investigated the association between school and APQ scores using multilevel linear regressions with APQ scores as the outcome and a random intercept for school. We calculated the intraclass correlation coefficient (ICC) to estimate the proportion of variance explained at school level. Next, we adjusted for potential individual- and school-level confounders.

We used samples with complete data on all variables. In a sensitivity analysis of the association with depressive symptoms, we imputed missing data on outcomes and confounders, using multiple imputation by chained equations (MICE).

## Results

### Sample Characteristics

Sample characteristics are described in Table S2. Mean scores on APQ items (possible range 0–4; see supplement pages 4–6) varied from 1.70 (SD = 1.27; item: ‘parental expectations’) to 2.88 (SD = 1.14; item: ‘worries about tests and exams’). Missingness on individual items ranged from 12.6% (item: ‘worries about getting a job’) to 17.1% (item: ‘teacher expectations’). The mean total APQ score was 15.24 (SD = 5.39, range 0–28, supplement, page 6).

### Factor Analysis

Of the 3,485 adolescents in sample 1, 2,316 had complete APQ data. There were no floor or ceiling effects (supplement, pages 4–6). Correlations between items ranged from r = 0.18 to r = 0.42 (supplement, page 11). Diagnostic criteria suggested data suitability for factor analysis (KMO = 0.84; Bartlett’s test of sphericity: Chi-squared = 3,155.58, df = 21, *p* < .0001). EFA suggested the extraction of one factor with an eigenvalue of 2.195, explaining 31.4% of the observed variance. The second largest eigenvalue was 0.163, suggesting this factor explained little variance. We therefore retained one factor (see scree plot supplement, page 12). Factor loadings ranged between 0.457 and 0.620, which suggests that all items are associated with the extracted factor (Table 2). The uniqueness of items was relatively high, ranging from 0.616 to 0.791 (Table 2). EFA results were unaltered in sensitivity analyses treating APQ items as ordinal and addressing missing data (supplement, pages 13–14).

**Table 2.** Factor Loadings and Uniqueness of Seven APQ Items (EFA and CFA)

	Factor loading EFA (CFA)	Uniqueness EFA (CFA)
Competition with peers for grades is intense	0.487 (0.526)	0.763 (0.723)
My parents' expectations about grades put me under pressure	0.611 (0.599)	0.626 (0.641)
There is pressure from teachers to perform well in tests and exams	0.585 (0.603)	0.658 (0.637)
I worry about doing well in tests or exams	0.620 (0.653)	0.616 (0.573)
I have too many tests and exams	0.457 (0.421)	0.791 (0.823)
If I fail to do well in school, I’m a failure as a person	0.590 (0.614)	0.652 (0.623)
Even if I do well in school, I’m worried about getting a job in the future	0.547 (0.582)	0.701 (0.661)

*Note.* Uniqueness describes the variance that is not explained by the extracted factor.

Of the 3,485 adolescents in sample 2 for the CFA (Table 2), 2,299 had complete data on the seven APQ items and were included in the analysis. Absolute and relative model fit were acceptable (Chi-squared = 192.58, *p* < .0001; RMSEA = 0.075, 95% CI: 0.065 to 0.084; SRMR = 0.037; CFI = 0.945; TLI = 0.917). CFA results were unaltered in sensitivity analyses treating APQ items as ordinal and addressing missing data (supplement, pages 15–16).

We found weak evidence of metric measurement invariance by sex and ethnicity. For sex and ethnicity, all three models (model 1: unrestricted; model 2: restricted to equal loadings; model 3: restricted to equal loadings and intercepts) had acceptable absolute and relative model fit (Supplemental Table S3). With each additional restriction, Chi-squared values increased, suggesting worse model fit, especially when groups were restricted to equal intercepts.

The factor analysis of the 10-item version showed similar results (supplement, pages 17–18).

### Reliability

Of the 6,970 adolescents, 4,615 had complete APQ data and were included in analyses. Cronbach’s alpha was 0.76 and was not substantially altered by removing items.

### Validity

#### Sex Differences

Male students (*n* = 2,113) had a mean APQ score of 14.06 (SD = 5.42) and female students (*n* = 2,472) of 16.42 (SD = 5.16). In the multilevel model, the mean difference was 1.98 (95% CI: 1.65 to 2.20).

Exploratory analyses of student and school characteristics revealed higher APQ scores among adolescents who were older or from minority-ethnic backgrounds and in schools with higher Ofsted ratings or higher Progress-8 scores (supplement page 20).

#### Association with Depressive Symptoms

Descriptive statistics are provided for the complete-case sample and according to level of the exposure in the supplement (page 19). We dropped one school with only one individual, leaving 2,379 individuals clustered within 40 schools. The minimum number of individuals per school was 19, the maximum 129 and the mean was 59.5 (SD = 28.8). The correlation between academic pressure scores and depressive symptoms was r = 0.51.

Estimates of all models are provided in the supplement (page 21). The univariable model showed strong evidence for an association between academic pressure and depressive symptoms (coefficient = 0.55, 95% CI: 0.51 to 0.59, *p* < .0001). Adjusting for confounders attenuated the association slightly (coefficient = 0.51, 95% CI: 0.48 to 0.55, *p* < .0001). Results were unaltered in sensitivity analyses that adjusted for mean school APQ and used multiple imputation (supplement, pages 21–22). Fully adjusted models provide evidence that the association was stronger among female than male students (*p* value for interaction < .0001; female students: coefficient = 0.65, 95% CI: 0.59 to 0.70, *p* < .0001; male students: coefficient = 0.37, 95% CI: 0.32 to 0.41, *p* < .0001).

#### Variation in Academic Pressure Between Schools

The mean school APQ was 15.26 (SD = 1.42) and ranged between 12.67 and 18.33. The ICC was 0.056 (95% CI: 0.033 to 0.096). After adjustment for individual characteristics, the ICC decreased to 0.037 (95% CI: 0.020 to 0.070). After additional adjustment for school variables, it decreased to 0.026 (95% CI: 0.01 to 0.06).

## Discussion

With young people from Wales (UK), we co-produced a short (7 item) but comprehensive measure of student perceptions of academic pressure, the APQ. The APQ is valid and reliable, so it can be used in research to investigate academic pressure in secondary-school adolescents and its associations with relevant potential causes (e.g. school-level factors) and outcomes (e.g. mental health or academic outcomes).

Our study has several limitations. Academic pressure is multidimensional and there are components and influences we did not measure. There is potential overlap between academic pressure, and symptoms of depression and anxiety. Our measure might have been affected by some of the same perceptual biases involved in depression and anxiety. However, we only found evidence of a moderate correlation between academic pressure scores and depressive symptoms (r = 0.51), which could be due to a potential causal association and suggests the constructs did not overlap substantially. Diversity within our young person’s advisory group was limited and the APQ might not capture the experiences of all students across the UK.

## Supplemental Material

Supplemental Material - Development of a Self-Reported Measure of Academic Pressure Among Secondary-School Students: The Academic Pressure Questionnaire Supplemental Material for Development of a Self-Reported Measure of Academic Pressure Among Secondary-School Students: The Academic Pressure Questionnaire by Marie A. E. Mueller, Chris Bonell, Tamsin J. Ford, Carolina Gutiérrez Muñoz, Ann John, Glyn Lewis, Rebecca Meiksin, Simon Murphy, George B. Ploubidis, Ruth Ponsford, Frances Rice, Thomas Steare, Alice Sullivan, Neisha Sundaram, Nerissa Tilouche and Gemma Lewis in Clinical Child Psychology and Psychiatry

## Data Availability

Data will be made available after the main trial analyses have been completed on reasonable request from researchers with ethics approval and a clear protocol.*
