# Supplementary material for: Development of a Self-Reported Measure of Academic Pressure Among Secondary-School Students: The Academic Pressure Questionnaire
Source: Clin Child Psychol Psychiatry. 2026 Mar 11;31(3):1126–35. doi: 10.1177/13591045261430414 (PMC13276109; doi:10.1177/13591045261430414)
Supplement: Supplemental Material - Development of a Self-Reported Measure of Academic Pressure Among Secondary-School Students: The Academic Pressure Questionnaire [file sj-pdf-1-ccp-10.1177_13591045261430414.pdf]

Supplementary material

Title: Development of a self-reported measure of academic pressure among secondary-school students: The Academic Pressure Questionnaire

Authors: Marie A. E. Mueller, Chris Bonell, Tamsin J. Ford, Carolina Gutiérrez Muñoz, Ann John, Glyn Lewis, Rebecca Meiksin, Simon Murphy, George Ploubidis, Ruth Ponsford, Frances Rice, Thomas Steare, Alice Sullivan, Neisha Sundaram, Nerissa Tilouche, Gemma Lewis

Corresponding author: Gemma Lewis (Division of Psychiatry, Faculty of Brain Sciences, University College London, London W1T 7NF, UK)

Measurement of confounders

Confounders included: age; family structure (lived with two parents; no/yes); and family affluence (6-item Family Affluence Scale III, ranging from 0 to 13; higher scores indicating higher affluence (Torsheim et al., 2016)). School-level confounders included the Income Deprivation Affecting Children Index (IDACI; measuring the proportion of children affected by income deprivation in the neighbourhood around the school), proportion of children eligible for free school meals, percentage of overall absence, proportion of children eligible for SEN support, proportion of children with English as an additional language, Ofsted rating (ranging from 1 'outstanding' to 3 'requires improvement'), Attainment 8 score (assessing the average academic performance of a school based on highest scores on eight government approved subjects), and Progress 8 score (assessing the progress students made between the end of primary school and the end of year 11, compared with the average progress in the country). Higher scores on the Attainment 8 score indicate higher attainment. Negative scores on the Progress 8 score suggest below-average progress, scores around zero suggest average progress, and positive scores suggest above-average progress.

Factor analysis methods

We randomly divided our sample into two, using the first sample for EFA and the second for CFA. We first assessed if items were suitable for EFA, investigating distributions, correlations, Kaiser-Meyer-Olkin (KMO) measure of sampling adequacy, and Bartlett's test of sphericity (Williams et al., 2010). We then extracted factors using principal axis factoring (PAF), a common estimation method in EFA (de Winter & Dodou, 2012). We assessed eigenvalues, scree plot, and percentage of explained variance. We considered content of factors, variance explained, and parsimony. To test if we could confirm factor structure, we ran a CFA. We tested whether the factor structure fitted the observed data of the second sample, using Structural Equation Modelling (SEM). In the main analysis, we included individuals with complete data on the seven APQ items. We tested measurement invariance for sex and ethnicity, using multi-group SEMs, assessing if extracted factors measured the same underlying latent construct across groups (Gregorich, n.d.). We ran models where all parameters could be freely estimated (Model 1), where loadings were restricted to be equal between groups (Model 2), and where both loadings and

intercepts were restricted to be equal between groups (Model 3). This deviated from the pre-registered protocol where we planned to run a multiple indicators multiple causes (MIMIC) model. After further consultation with collaborators, we decided to run multi-group SEMs because they allow for more rigorous testing of invariance. MIMIC models assume the invariance of factor loadings between groups and estimate only one set of loadings (Kim et al., 2012).

For EFA, we ran two sensitivity analyses. First, we treated APQ items as ordinal (instead of continuous). Second, we addressed missing data. Multiple Imputation commands do not work with Stata’s factor commands. We therefore used maximum likelihood with the expectation-maximization (EM) algorithm to estimate the covariance matrix of items and ran a factor analysis using this matrix. For CFA, we ran two sensitivity analyses. First, we treated APQ items as ordinal. Second, we imputed missing APQ item data, using multivariate normal regression and 50 imputations. We then ran a CFA (using SEM) on imputed data.

In a supplementary analysis, we ran EFA and CFA for the 10-item version of the APQ.

Distributions of responses to APQ items

Competition with peers for grades is intense

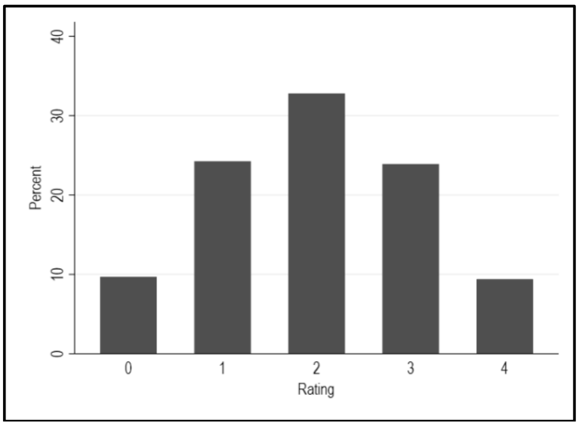

My parents' expectations about grades put me under pressure

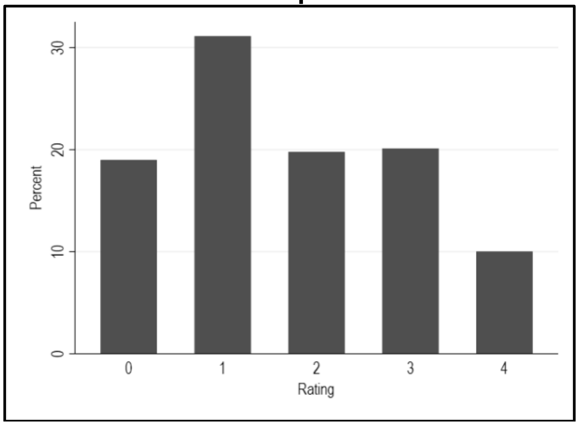

There is pressure from teachers to perform well in tests and exams

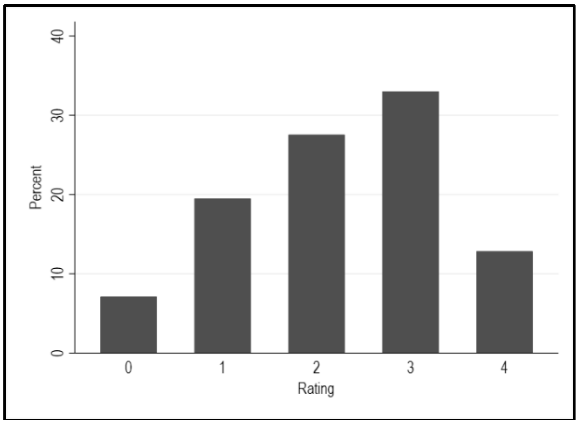

I worry about doing well in tests or exams

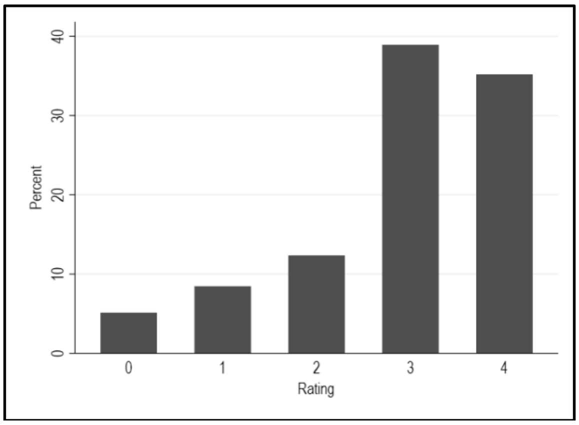

I have too many tests and exams

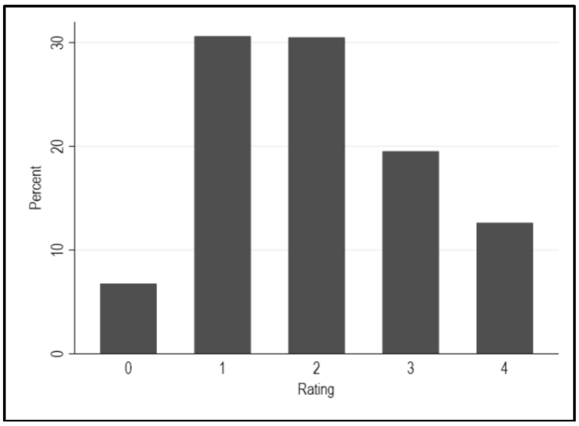

If I fail to do well in school, I'm a failure as a person

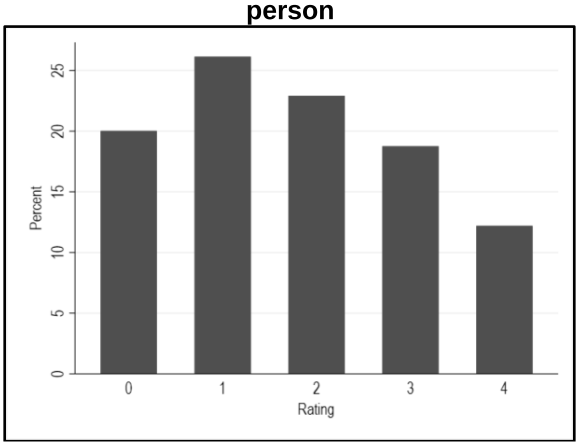

Even if I do well in school, I'm worried about getting a job in the future

My school sets too much homework [1]

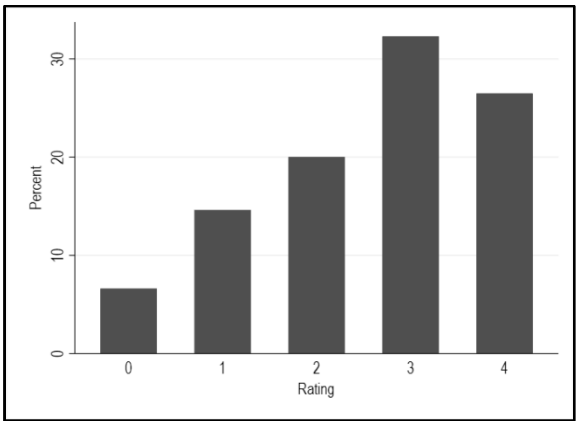

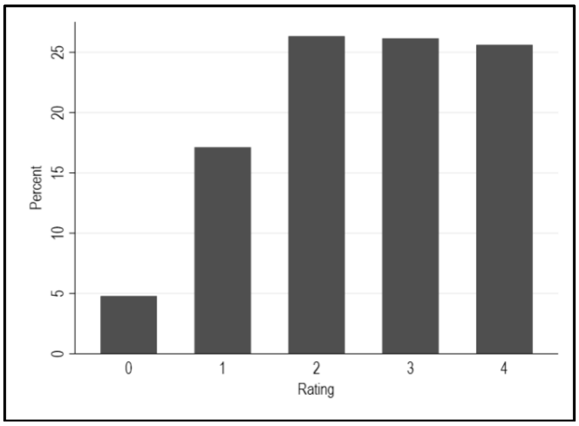

I'm confident I will live up to my academic standards [1, 2]

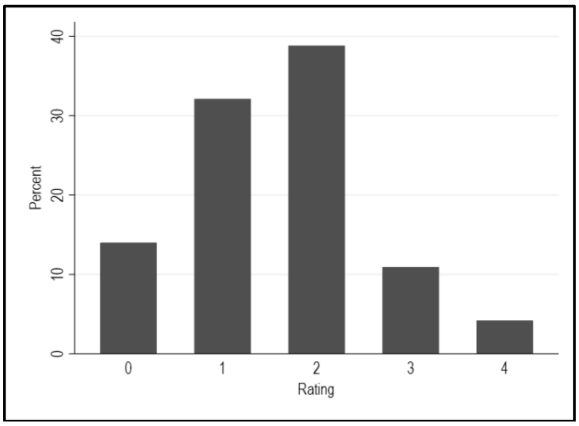

My grades are important to my future and might even determine my whole life [1]

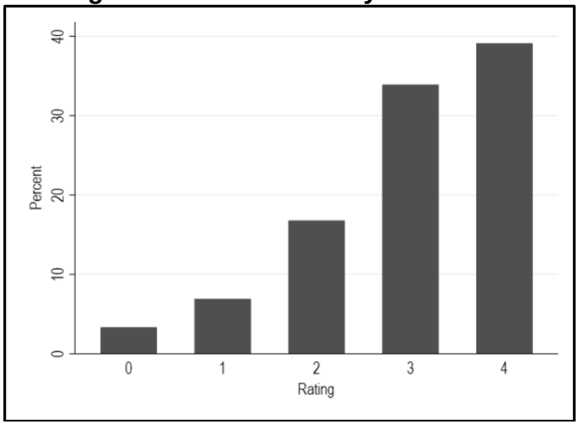

Note: [1] These items are removed from the 7-item APQ. [2] This item is reverse coded to 0 'strongly agree' and 4 'strongly disagree'. All other items are coded 0 'strongly disagree' to 4 'strongly agree'.

Distribution of total APQ scores

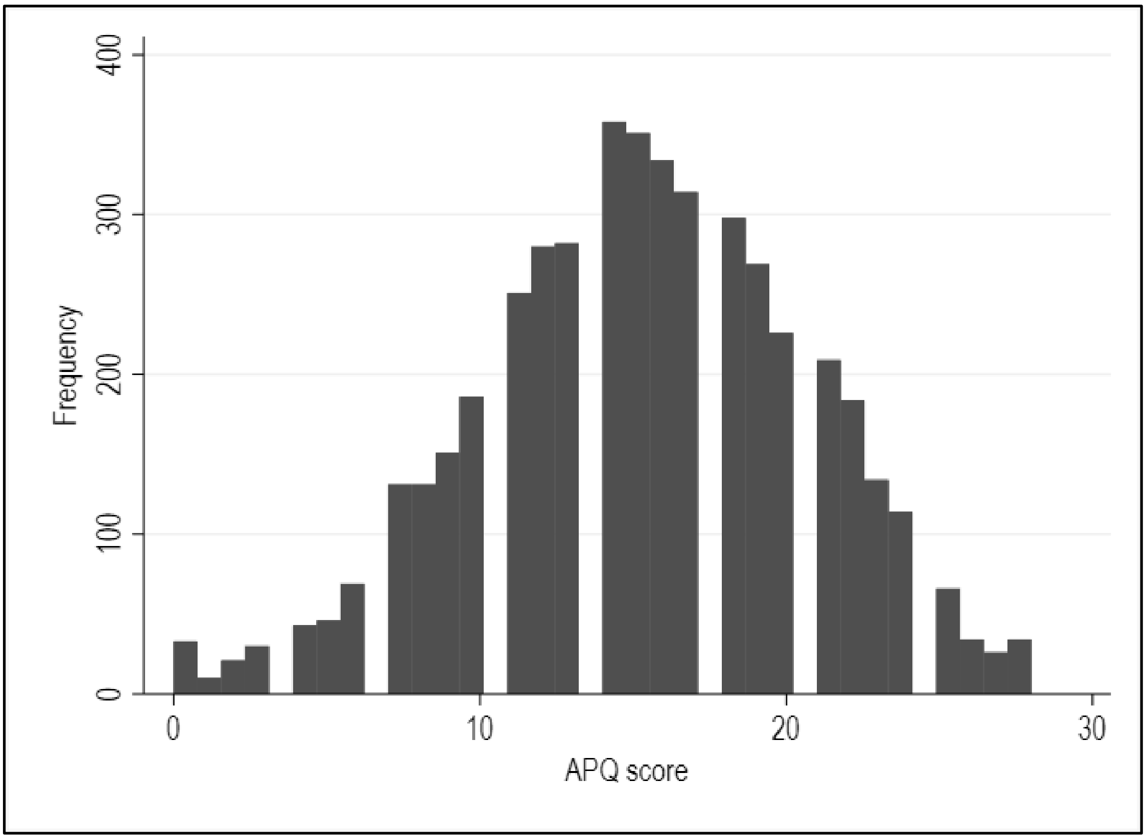

Note: Histogram of APQ scores (n = 4,615). APQ scores can range between 0 and 28. The mean APQ score was 15.24 (SD = 5.39).

Table S1
Existing validated measures of academic pressure or related constructs for secondary-school students

Measure	Dimensions	Number of items	Sample	Country	Initial validation [5]	Main limitations
Adolescent Stress Questionnaire (ASQ)	Stress of home life, school performance, school attendance, romantic relationships, peer pressure, teacher interaction, future uncertainty, school/leisure conflict, financial pressure, emerging adult responsibility.	58	High school students (years 7 to 10) and secondary college students (school years 11 and 12).	Australia	Concurrent criterion validity: Positive associations with anxiety and depression, and a negative association with self-esteem.	Large number of items. Large number of dimensions not limited to academic pressure.
Short version of the Adolescent Stress Questionnaire (ASQ-S)	Stress of home life, school performance, school attendance, romantic relationships, peer pressure, teacher interaction, future uncertainty, school/leisure conflict, financial pressure.	27	Secondary-school students (Grades 7 and 8).	Sweden	Concurrent and predictive validity: Positive associations for some subscales with anxiety, depression, and worry, and a negative association with self-esteem.	Relatively large number of items. Large number of dimensions not limited to academic pressure.
14-item Adolescent Stress Questionnaire (ASQ-14)	One factor measuring adolescent stress.	14	Ages 12 to 18 years.	Spain	Positive correlations with stress manifestations, anxiety, depression, and emotional and behavioural problems, and a negative correlation with life satisfaction.	Items measuring adolescent stress more generally but not limited to academic pressure.
Academic Expectations Stress Inventory (AESI)	Parent- and teacher expectations, self-expectations.	9 [1]	Secondary-school students (Grades 7 to 10, ages 12 to 18).	Singapore	Convergent validity: Association with anxiety. Discriminant validity: No association with sensation seeking or self-reliance.	Limited to academic expectations. No inclusion of adolescents in the development of items.
Educational Stress Scale for Adolescents (ESSA)	Pressure from study, workload, worry about grades, self-expectation, despondency.	16 [2]	Secondary-school students (Grades 7 to 12, ages 11 to 20).	China	Concurrent validity: Association with AESI. Predictive validity: Associations with academic grades, depressive symptoms, and suicidal thoughts.	Relatively large number of items. No inclusion of adolescents in the development of items.
Iranian Students' Academic Stress Questionnaire (IAASQ)	Stress of home life, educational system, future uncertainty, academic competition, teacher interaction, school regulations, peer pressure, parents involved, financial pressure.	57 [3]	Secondary-school students (ages 12 to 18 years).	Iran	Content validity: The initial version of the IAASQ was reviewed by 'content' experts and 'lay' experts. Construct validity: Associations with self-efficacy and mental health difficulties.	Large number of items. Large number of dimensions not limited to academic pressure. Limited inclusion of adolescents in the development of items.
Academic Stress in Secondary Education (QASSE)	Academic overload, interactions with classmates, family pressure, future perspectives.	24 [4]	Secondary-school students (Grades 7 to 12, mean age 14.6 years).	Spain	Convergent validity: Associations with mental health and somatic symptoms.	Large number of items. Limited inclusion of adolescents in the development of items.

Note: These measures have been developed for secondary-school students. Studies have attempted validation of these measures. [1] After removal of 6 items. [2] After removal of 14 items. [3] After removal of 18 items. [4] Included 30 items but only 24 contributed to the four extracted dimensions. [5] Some measures have been validated further (e.g. in other countries).

Table S2
Sample characteristics (N = 6,970)

		Full sample (N = 6,970)	Sample 1 (n = 3,485)	Sample 2 (n = 3,485)
		**n (%)**	**n (%)**	**n (%)**
Age				
	12	3,797 (54.5)	1,922 (55.2)	1,875 (53.8)
	13	3,101 (44.5)	1,518 (43.6)	1,583 (45.4)
	Missing	72 (1.0)	45 (1.3)	27 (0.8)
Sex				
	Male	3,291 (47.2)	1,658 (47.6)	1,633 (46.9)
	Female	3,621 (52.0)	1,796 (51.5)	1,825 (52.4)
	Missing	58 (0.8)	31 (0.9)	27 (0.8)
Ethnicity				
	Asian or Asian British	458 (6.6)	231 (6.6)	227 (6.5)
	Black African, Black Caribbean, or Black	392 (5.6)	185 (5.3)	207 (5.9)
	Mixed/multiple ethnic groups	396 (5.7)	183 (5.3)	213 (6.1)
	White	4,833 (69.3)	2,444 (70.1)	2,389 (68.6)
	Any other ethnic group	86 (1.2)	45 (1.3)	41 (1.2)
	Missing	805 (11.6)	397 (11.4)	408 (11.7)
Lives with two parents				
	No	1,320 (18.9)	672 (19.3)	648 (18.6)
	Yes	5,543 (79.5)	2,763 (79.3)	2,780 (79.8)
	Missing	107 (1.5)	50 (1.4)	57 (1.6)
Ofsted rating				
	Outstanding	1,171 (16.8)	572 (16.4)	599 (17.2)
	Good	4,379 (62.8)	2,180 (62.6)	2,199 (63.1)
	Requires improvement	551 (7.9)	298 (8.6)	253 (7.3)
	Missing	869 (12.5)	435 (12.5)	434 (12.5)
		**Mean (SD)**	**Mean (SD)**	**Mean (SD)**
PHQ-8		5.85 (5.94)	5.85 (5.92)	5.85 (5.96)
	Missing (n [%])	2,074 (29.8)	1,052 (30.2)	1,022 (29.3)
Competition with peers for grades is intense		1.97 (1.12)	1.99 (1.12)	1.94 (1.12)
	Missing (n [%])	1,021 (14.7)	514 (14.8)	507 (14.6)
My parents' expectations about grades put me under pressure		1.70 (1.27)	1.71 (1.26)	1.69 (1.28)
	Missing (n [%])	1,130 (16.2)	547 (15.7)	583 (16.7)
There is pressure from teachers to perform well in tests and exams		2.23 (1.13)	2.25 (1.12)	2.21 (1.14)
	Missing (n [%])	1,191 (17.1)	595 (17.1)	596 (17.1)
I worry about doing well in tests or exams		2.88 (1.14)	2.91 (1.13)	2.86 (1.15)
	Missing (n [%])	1,020 (14.6)	517 (14.8)	503 (14.4)
I have too many tests and exams		2.00 (1.12)	2.01 (1.13)	2.00 (1.12)
	Missing (n [%])	1,014 (14.6)	512 (14.7)	502 (14.4)
If I fail to do well in school, I'm a failure as a person		1.77 (1.30)	1.77 (1.30)	1.77 (1.29)
	Missing (n [%])	1,096 (15.7)	573 (16.4)	523 (15.0)
Even if I do well in school, I'm worried about getting a job in the future		2.57 (1.21)	2.57 (1.21)	2.56 (1.22)
	Missing (n [%])	877 (12.6)	446 (12.8)	431 (12.4)

Academic pressure score	15.24 (5.39)	15.35 (5.35)	15.13 (5.43)
Missing (n [%])	2,355 (33.8)	1,169 (33.5)	1,186 (34.0)
FAS	6.87 (1.64)	6.89 (1.62)	6.85 (1.65)
Missing (n [%])	1,148 (16.5)	585 (16.8)	563 (16.2)
IDACI	0.12 (0.10)	0.12 (0.10)	0.12 (0.10)
Missing (n [%])	0 (0)	0 (0)	0 (0)
Proportion of children eligible for free school meals	0.21 (0.14)	0.21 (0.14)	0.21 (0.14)
Missing (n [%])	67 (1.0)	32 (0.9)	35 (1.0)
Percentage of overall absence	5.45 (1.11)	5.48 (1.12)	5.42 (1.10)
Missing (n [%])	217 (3.1)	108 (3.1)	109 (3.1)
Proportion of children eligible for SEN support	0.13 (0.05)	0.12 (0.05)	0.13 (0.05)
Missing (n [%])	0 (0)	0 (0)	0 (0)
Proportion of children with English as an additional language	0.15 (0.15)	0.15 (0.15)	0.15 (0.15)
Missing (n [%])	0 (0)	0 (0)	0 (0)
Attainment 8 score	48.74 (9.39)	48.62 (9.43)	48.86 (9.35)
Missing (n [%])	264 (3.8)	132 (3.8)	132 (3.8)
Progress 8 score	0.04 (0.42)	0.03 (0.42)	0.05 (0.42)
Missing (n [%])	331 (4.8)	164 (4.7)	167 (4.8)

Note: The full sample was split randomly into sample 1 and sample 2. Age group “13” includes a small number of 14-year-olds.

Table S3
Model fit indices for multigroup SEMs by sex and ethnicity

	Chi-squared	RMSEA [95% CI]	SRMR	CFI	TLI
Sex					
Model 1	385.62 *	0.075 [0.068, 0.081]	0.038	0.941	0.912
Model 2	410.32 *	0.069 [0.064, 0.076]	0.043	0.938	0.923
Model 3	581.96 *	0.077 [0.071, 0.082]	0.046	0.911	0.906
Ethnicity					
Model 1	330.56 *	0.072 [0.066, 0.079]	0.039	0.947	0.920
Model 2	342.55 *	0.066 [0.060, 0.073]	0.044	0.946	0.933
Model 3	506.38 *	0.075 [0.069, 0.081]	0.044	0.918	0.914

Note: Model 1: unrestricted; Model 2: restricted to equal loadings; Model 3: restricted to equal loadings and intercepts. RMSEA = Root Mean Square Error of Approximation; SRMR = Standardized Root Mean Squared Residual, CFI = Comparative Fit Index, TLI = Tucker-Lewis Index. * $p < 0.0001$.

Correlations between APQ items

Table S4
Correlations between APQ items

	1	2	3	4	5	6	7	8 [1]	9 [1, 2]	10 [1]
1	-									
2	0.343	-								
3	0.313	0.391	-							
4	0.308	0.346	0.364	-						
5	0.217	0.267	0.370	0.249	-					
6	0.249	0.403	0.286	0.389	0.243	-				
7	0.217	0.309	0.276	0.442	0.183	0.416	-			
[1] 8	0.137	0.165	0.328	0.163	0.548	0.148	0.120	-		
[1, 2] 9	-0.017	0.120	0.144	0.146	0.060	0.222	0.215	0.065	-	
[1] 10	0.208	0.239	0.117	0.277	0.079	0.358	0.356	0.012	-0.079	-

Note: [1] These items are removed from the 7-item APQ. [2] This item is reverse coded to 0 ‘strongly agree’ and 4 ‘strongly disagree’. All other items are coded 0 ‘strongly disagree’ to 4 ‘strongly agree’.

Item 1: Competition with peers for grades is intense
Item 2: My parents' expectations about grades put me under pressure
Item 3: There is pressure from teachers to perform well in tests and exams
Item 4: I worry about doing well in tests or exams
Item 5: I have too many tests and exams
Item 6: If I fail to do well in school, I'm a failure as a person
Item 7: Even if I do well in school, I'm worried about getting a job in the future
Item 8: My school sets too much homework [1]
Item 9: I'm confident I will live up to my academic standards [1]
Item 10: My grades are important to my future and might even determine my whole life [1]

Scree plot

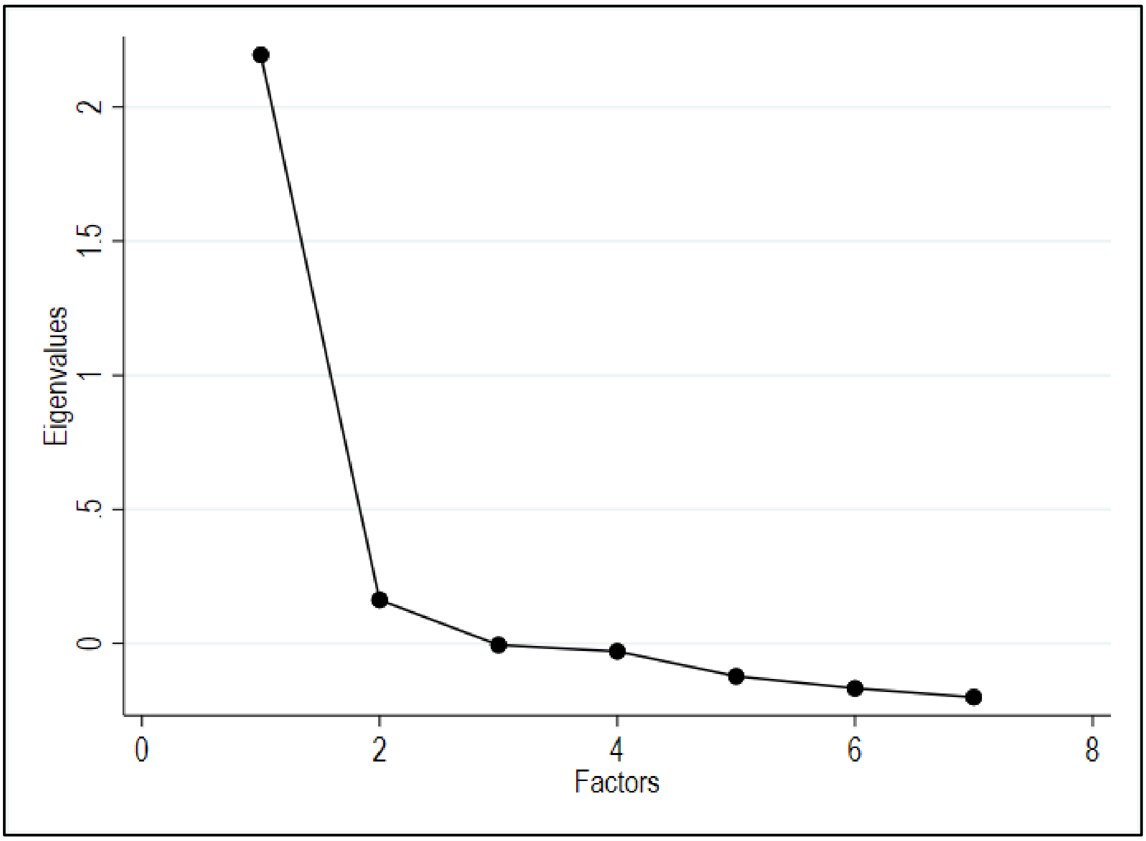

EFA using ordinal data

Ordinal EFA suggested the extraction of three factors with positive eigenvalues of 2.561, 0.198, and 0.000. As factors two and three did not explain significantly more variance, we extracted only the first factor, explaining 36.6% of the observed variance of the seven items. Results do not differ substantially from the main analysis (treating items as continuous).

Table S5
Factor loadings and uniqueness of seven APQ items (taken from ordinal EFA)

	Factor loading	Uniqueness
Competition with peers for grades is intense	0.517	0.733
My parents' expectations about grades put me under pressure	0.651	0.576
There is pressure from teachers to perform well in tests and exams	0.624	0.610
I worry about doing well in tests or exams	0.697	0.514
I have too many tests and exams	0.495	0.755
If I fail to do well in school, I'm a failure as a person	0.631	0.602
Even if I do well in school, I'm worried about getting a job in the future	0.593	0.649

Note: Uniqueness describes the variance that is not explained by the extracted factor.

EFA using the covariance matrix of APQ items

EFA of the covariance matrix of the seven APQ items suggested the extraction of two factors with positive eigenvalues of 2.154 and 0.194. As factor two did not explain significantly more variance, we extracted only one factor, explaining 30.8% of the observed variance of the seven items. Results do not differ substantially from the main analysis (using complete data).

Table S6
Factor loadings and uniqueness of seven APQ items (taken from EFA using the covariance matrix of APQ items)

	Factor loading	Uniqueness
Competition with peers for grades is intense	0.476	0.773
My parents' expectations about grades put me under pressure	0.600	0.640
There is pressure from teachers to perform well in tests and exams	0.577	0.667
I worry about doing well in tests or exams	0.617	0.620
I have too many tests and exams	0.441	0.805
If I fail to do well in school, I'm a failure as a person	0.590	0.652
Even if I do well in school, I'm worried about getting a job in the future	0.558	0.689

Note: Uniqueness describes the variance that is not explained by the extracted factor.

CFA using ordinal data

Using CFA with ordinal data, results were comparable to results from CFA with continuous data. To run CFA with ordinal data, we used generalised SEM ("gsem") in Stata, using the "oprobit" option. The "gsem" function in Stata does not provide standardized coefficients, so we provide unstandardized coefficients below. We do not present error variances, as these are not available for "gsem" with ordinal data.

Table S7
Unstandardized coefficients and error variances for CFA using continuous and ordinal data

	Continuous data Coefficient	Ordinal data Coefficient
Competition with peers for grades is intense	1.000	1.000
My parents' expectations about grades put me under pressure	1.314	1.254
There is pressure from teachers to perform well in tests and exams	1.172	1.236
I worry about doing well in tests or exams	1.282	1.572
I have too many tests and exams	0.804	0.753
If I fail to do well in school, I'm a failure as a person	1.359	1.292
Even if I do well in school, I'm worried about getting a job in the future	1.214	1.197

CFA using imputed data

Running CFA using imputed data, results were comparable to results from CFA using complete data. SEM is not officially supported by “mi estimate” in Stata. We ran it using the “cmdok” option, but we were not able to produce standardized coefficients. Below, we present unstandardized coefficients from SEMs using complete and imputed data to allow for the comparison of results. Unstandardized coefficients and error variances are similar for complete and imputed data.

Table S8

Unstandardized coefficients and error variances for CFA using complete and imputed data

	Complete data Coefficient	Complete data Error variance	Imputed data Coefficient	Imputed data Error variance
Competition with peers for grades is intense	1.000	0.898	1.000	0.949
My parents' expectations about grades put me under pressure	1.314	1.062	1.338	1.060
There is pressure from teachers to perform well in tests and exams	1.172	0.829	1.172	0.864
I worry about doing well in tests or exams	1.282	0.760	1.340	0.745
I have too many tests and exams	0.804	1.034	0.811	1.042
If I fail to do well in school, I'm a failure as a person	1.359	1.052	1.411	1.033
Even if I do well in school, I'm worried about getting a job in the future	1.214	0.988	1.256	0.978

Note: Uniqueness describes the variance that is not explained by the extracted factor. Missing data were imputed using multivariate normal regression and 50 imputations. N for complete data analysis = 2,299. N for imputed data analysis = 3,485.

Factor analysis for the 10-item APQ

Indicators of sampling adequacy

- KMO = 0.789
- Bartlett's test of sphericity: Chi-squared = 4492.305, dfs = 45, $p < 0.0001$

Scree plot

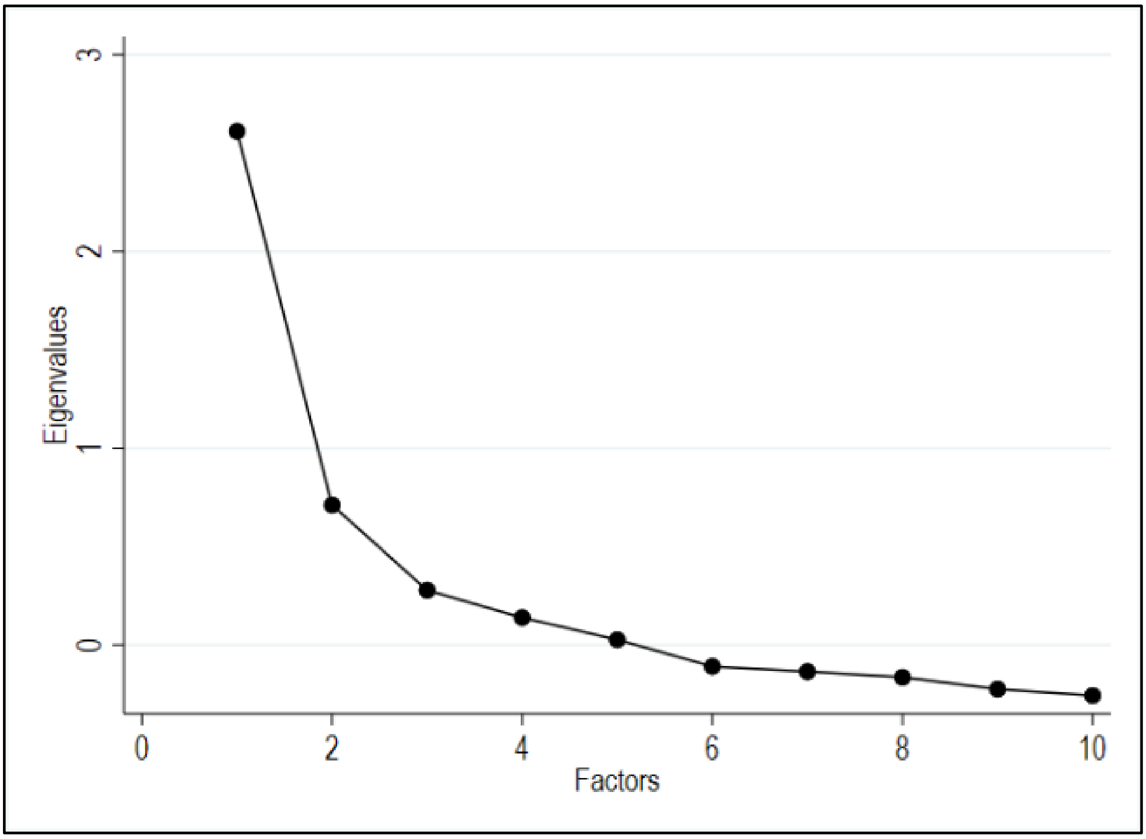

Factor loadings

Table S9
Factor loadings and uniqueness of ten APQ items (EFA and CFA)

	Factor loading EFA (CFA)	Uniqueness EFA (CFA)
Competition with peers for grades is intense	0.478 (0.493)	0.774 (0.757)
My parents' expectations about grades put me under pressure	0.588 (0.581)	0.655 (0.663)
There is pressure from teachers to perform well in tests and exams	0.584 (0.587)	0.659 (0.655)
I worry about doing well in tests or exams	0.608 (0.629)	0.631 (0.604)
I have too many tests and exams	0.520 (0.445)	0.729 (0.802)
If I fail to do well in school, I'm a failure as a person	0.616 (0.624)	0.621 (0.611)
Even if I do well in school, I'm worried about getting a job in the future	0.558 (0.600)	0.689 (0.640)
My school sets too much homework	0.395 (0.348)	0.844 (0.879)
I'm confident I will live up to my academic standards	0.200 (0.270)	0.960 (0.927)
My grades are important to my future and might even determine my whole life	0.415 (0.427)	0.827 (0.818)

Note: Uniqueness describes the variance that is not explained by the extracted factor.

CFA fit indices

- Chi-squared = 1051.789, dfs = 35, $p < 0.0001$
- RMSEA = 0.118, 95% CI: 0.112 to 0.124
- SRMR = 0.077
- CFI = 0.766
- TLI = 0.699

Cronbach’s alpha

- Cronbach’s alpha = 0.77

Sample characteristics of the complete-case sample

Table S10
Characteristics of complete-case sample (n = 2,379)

	Full sample (n = 2,379)	Low APQ (n = 1,251)	High APQ (n = 1,128)
	**n (%)** [1]	**n (%)** [2]	**n (%)** [2]
Age			
12	1,282 (53.9)	698 (54.45)	584 (45.55)
13	1,097 (46.1)	553 (50.41)	544 (49.59)
Sex			
Male	1,029 (43.4)	668 (64.92)	361 (35.08)
Female	1,350 (56.8)	583 (43.19)	767 (56.81)
Ethnicity			
Asian or Asian British	171 (7.2)	76 (44.44)	95 (55.56)
Black African, Black Caribbean, or Black	146 (6.1)	57 (39.04)	89 (60.96)
Mixed/multiple ethnic groups	152 (6.4)	71 (46.71)	81 (53.29)
White	1,882 (79.1)	1,047 (54.82) [3]	863 (45.18) [3]
Any other ethnic group	28 (1.2)		
Lives with two parents			
No	398 (16.7)	200 (50.25)	198 (49.75)
Yes	1,981 (83.3)	1,051 (53.05)	930 (46.95)
Ofsted rating			
Outstanding	564 (23.7)	250 (44.33)	314 (55.67)
Good	1,590 (66.8)	865 (54.40)	725 (45.60)
Requires improvement	225 (9.5)	136 (60.44)	89 (39.56)
	**Mean (SD)**	**Mean (SD)**	**Mean (SD)**
PHQ-8	6.16 (6.01)	3.56 (4.38)	9.04 (6.25)
Academic pressure score	15.01 (5.45)	10.88 (3.53)	19.60 (2.97)
FAS	6.86 (1.60)	6.85 (1.56)	6.87 (1.64)
IDACI	0.12 (0.10)	0.12 (0.10)	0.13 (0.10)
Proportion of children eligible for free school meals	0.20 (0.14)	0.20 (0.13)	0.20 (0.15)
Percentage of overall absence	5.33 (1.14)	5.39 (1.07)	5.28 (1.22)
Proportion of children eligible for SEN support	0.13 (0.05)	0.13 (0.05)	0.13 (0.06)
Proportion of children with English as an additional language	0.15 (0.14)	0.14 (0.14)	0.16 (0.14)
Attainment 8 score	49.82 (9.65)	48.79 (8.84)	50.97 (10.35)
Progress 8 score	0.06 (0.43)	0.02 (0.41)	0.11 (0.45)

Note: Age group "13" includes a small number of 14-year-olds. [1] Column percentages. [2] Row percentages. [3] Due to cell sizes < 20, to protect anonymity, we combined 'White' and 'Any other ethnic group' for by-exposure statistics in this table.

Exploratory analyses of characteristics associated with academic pressure

**Table S11**<br>Complete-case sample (n = 2,379)			
	**Mean Difference**	**95% CI**	**P value**
Age			
12	ref	ref	ref
13	0.37	0.05 to 0.68	0.021
Ethnicity			
White	ref	ref	ref
Asian or Asian British	0.93	0.31 to 1.54	0.003
Black African, Black Caribbean, or Black	0.82	0.09 to 1.54	0.027
Mixed/multiple ethnic groups	0.64	-0.05 to 1.33	0.068
Any other ethnic group	0.85	-0.69 to 2.39	0.28
Lives with two parents			
No	ref	ref	ref
Yes	-0.26	-0.68 to 0.15	0.21
Ofsted rating			
Requires improvement	ref	ref	ref
Good	-0.95	-2.01 to 0.10	0.08
Outstanding	-1.47	-3.07 to 0.13	0.07
	**Coefficient**	**95% CI**	**P value**
FAS	-0.02	-0.12 to 0.08	0.71
IDACI	2.96	-0.79 to 6.70	0.12
Proportion of children eligible for free school meals	0.93	-1.73 to 3.59	0.49
Percentage of overall absence	-0.13	-0.48 to 0.21	0.45
Proportion of children eligible for SEN support	-1.78	-9.32 to 5.76	0.64
Proportion of children with English as an additional language	1.62	-0.64 to 3.88	0.16
Attainment 8 score	0.03	-0.01 to 0.07	0.13
Progress 8 score	1.06	0.19 to 1.93	0.02

Association between academic pressure and depressive symptoms

Table S12
Estimates for models with individual APQ score as the exposure (n = 2,379)

	Coefficient	95% CI	p value
Model 1	0.55	0.51 to 0.59	< 0.0001
Model 2	0.51	0.47 to 0.55	< 0.0001
Model 3	0.51	0.47 to 0.55	< 0.0001
Model 4.1	0.51	0.47 to 0.55	< 0.0001
Model 4.2	0.51	0.47 to 0.55	< 0.0001
Model 4.3	0.51	0.47 to 0.55	< 0.0001
Model 4.4	0.51	0.47 to 0.55	< 0.0001
Model 4.5	0.51	0.47 to 0.55	< 0.0001
Model 4.6	0.51	0.48 to 0.55	< 0.0001
Model 4.7	0.51	0.48 to 0.55	< 0.0001
Model 4.8	0.51	0.48 to 0.55	< 0.0001
Model 5	0.51	0.48 to 0.55	< 0.0001
Model 6	0.51	0.47 to 0.55	< 0.0001

Note:

Model 1: Univariable model
Model 2: Model 1 + individual-level demographic variables
Model 3: Model 2 + family structure and FAS
Model 4: Model 3 + separate adjustments for

- IDACI (4.1)
- Proportion of children eligible for free school meals (4.2)
- Percentage of overall absence (4.3)
- Proportion of children eligible for SEN support (4.4)
- Proportion of children with English as an additional language (4.5)
- Ofsted rating (4.6)
- Attainment 8 score (4.7)
- Progress 8 score (4.8)

Model 5: Model 3 + all school-level confounders
Model 6: Model 5 + mean school APQ score

Multiple imputation estimates

Table S13

Multiple imputation estimates for models with individual APQ score as the exposure (n = 4,613)

	Coefficient	95% CI	p value
Model 1	0.55	0.52 to 0.58	< 0.0001
Model 2	0.52	0.48 to 0.55	< 0.0001
Model 3	0.52	0.48 to 0.55	< 0.0001
Model 4.1	0.51	0.48 to 0.55	< 0.0001
Model 4.2	0.52	0.48 to 0.55	< 0.0001
Model 4.3	0.52	0.48 to 0.55	< 0.0001
Model 4.4	0.52	0.48 to 0.55	< 0.0001
Model 4.5	0.52	0.48 to 0.55	< 0.0001
Model 4.6	0.52	0.49 to 0.55	< 0.0001
Model 4.7	0.52	0.49 to 0.55	< 0.0001
Model 4.8	0.52	0.49 to 0.55	< 0.0001
Model 5	0.52	0.49 to 0.55	< 0.0001
Model 6	0.52	0.48 to 0.55	< 0.0001

Note: Estimates are pooled estimates from 50 imputations.

Model 1: univariable model
Model 2: Model 1 + individual-level demographic variables
Model 3: Model 2 + family structure and FAS
Model 4: Model 3 + separate adjustments for

- IDACI (4.1)
- Proportion of children eligible for free school meals (4.2)
- Percentage of overall absence (4.3)
- Proportion of children eligible for SEN support (4.4)
- Proportion of children with English as an additional language (4.5)
- Ofsted rating (4.6)
- Attainment 8 score (4.7)
- Progress 8 score (4.8)

Model 5: Model 3 + all school-level confounders
Model 6: Model 5 + mean school APQ score

References

de Winter, J. C. F., & Dodou, D. (2012). Factor recovery by principal axis factoring and maximum likelihood factor analysis as a function of factor pattern and sample size. *Journal of Applied Statistics*, *39*(4), 695–710. https://doi.org/10.1080/02664763.2011.610445

Gregorich, S. E. (n.d.). *Do Self-Report Instruments Allow Meaningful Comparisons Across Diverse Population Groups? Testing Measurement Invariance Using the Confirmatory Factor Analysis Framework*.

Kim, E. S., Yoon, M., & Lee, T. (2012). Testing Measurement Invariance Using MIMIC: Likelihood Ratio Test With a Critical Value Adjustment. *Educational and Psychological Measurement*, *72*(3), 469–492. https://doi.org/10.1177/0013164411427395

Torsheim, T., Cavallo, F., Levin, K. A., Schnohr, C., Mazur, J., Niclasen, B., & Currie, C. (2016). Psychometric Validation of the Revised Family Affluence Scale: a Latent Variable Approach. *Child Indicators Research*, *9*(3), 771–784. https://doi.org/10.1007/S12187-015-9339-X

Williams, B., Onsman, A., Brown, T., Andrys Onsman, P., & Ted Brown, P. (2010). Exploratory Factor Analysis: A Five-Step Guide for Novices. *Https://Doi.Org/10.33151/Ajp.8.3.93*, *8*(3), 1–13. https://doi.org/10.33151/AJP.8.3.93
